# In Vitro Resistance of Natural Molars vs. Additive-Manufactured Simulators Treated with Pulpotomy and Endocrown

**DOI:** 10.3390/jfb14090444

**Published:** 2023-08-29

**Authors:** Marie-Laure Munoz-Sanchez, Alexis Gravier, Olivier Francois, Emmanuel Nicolas, Martine Hennequin, Nicolas Decerle

**Affiliations:** 1 Centre de Recherche en Odontologie Clinique (CROC), Université Clermont Auvergne, F-63000 Clermont-Ferrand, France; m-laure.munoz-sanchez@uca.fr (M.-L.M.-S.); olivier.francois@uca.fr (O.F.); martine.hennequin@uca.fr (M.H.); nicolas.decerle@uca.fr (N.D.); 2 CHU Clermont-Ferrand, Service d’Odontologie, F-63003 Clermont-Ferrand, France; 3 Institut Pascal UMR CNRS 6602, Université Clermont Auvergne, F-63000 Clermont-Ferrand, France; alexis.gravier@sigma-clermont.fr

**Keywords:** endodontically treated teeth, pulpotomy, endocrown, fracture strength

## Abstract

Endocrowns are designed to restore endodontically treated teeth with root canal treatment (Rct). Recently, endocrowns were proposed for teeth treated with full pulpotomy (FP). No data exist on in vitro evaluations for this combination. This study aimed to evaluate the mechanical behavior of pulpotomy-treated teeth with endocrowns according to different protocols for preparation design and materials and to assess whether 3D-printed resin simulators could be a reliable alternative for human teeth during in vitro strength tests. One hundred and ten extracted natural molars were randomized into 11 groups according to the type of endodontic treatment, the material used, and the design of peripheric preparation. One hundred and ten resin simulators were separated similarly. The samples were embedded in epoxy resin blocks before being subjected to oblique compressive load until failure. For natural teeth, the variance analysis separated two homogeneous groups, one regrouping the endodontically treated or pulpotomy-treated teeth without coronal restoration and the other one regrouping all the other samples, i.e., the untreated teeth (positive controls) and the treated and restored teeth. The strength resistance was lower for the resin simulators than for natural teeth in all groups. Within the limit of this study, strength resistance is not the most important criterion for choosing the type of material, preparation, or endodontic treatment for endocrowns. Resin simulators are not efficient for in vitro strength studies.

## 1. Introduction

Restoring endodontically treated teeth has long been a biological and biomechanical challenge [1]. Factors affecting the outcome of coronal restoration have been investigated extensively. The extent of coronal tissue loss, the use of a root anchorage, and the design of the peripheral limit, particularly with or without a circumferential ferrule, are still topics of discussion [2]. Until recently, the dehydration of treated teeth [3,4] and/or additional loss of tissues due to the canal preparation [5] were hypotheses used to explain long-term failures due to root fractures. During the last decade, the evolution of CAD/CAM procedures and pulp regeneration therapeutics has led to new solutions for the restoration of endodontically treated teeth. It has already been reported that CAD/CAM endocrown restorations could be performed on posterior teeth with root canal treatment (Rct), a solution that has been assessed as having a low risk of failure after several years [6,7,8]. It was also reported that CAD/CAM endocrowns could be safely recommended to restore severely damaged permanent molars with pulpitis treated with full pulpotomy (FP) [9]. The combination of an endocrown and full pulpotomy has the advantage of preserving the residual biological potential of the pulp and ensuring an endo-prosthetic continuum with the objective of long-term success. Before carrying out further clinical trials, in vitro assessments are needed to base clinical procedures on evidence. Three points should be taken into consideration: (I) It is unknown whether the mechanical behavior of endocrowns differs between teeth being treated with full pulpotomy or root canal treatment. (II) The design of the coronal preparation has already been investigated, but the results are still being debated [2]. In particular, additional experiments comparing coronal preparation with or without a peripheral ferrule are needed. (III) Contradictory results have been reported on the relative resistance of both composite and ceramic materials used for endocrown milling [10].

This study has a supplementary goal that concerns the interest of using 3D-printed resin simulators rather than human teeth for in vitro experiments. Collecting sound human molars with the same anatomical characteristics [11] for in vitro studies as well as for educational issues is difficult. Moreover, the first and second molars collected for studies are often damaged before being extracted due to the carious process or restorations, and the third molars are often cut during extraction to preserve most of the bone. The dental community would certainly benefit from using validated simulators instead of natural human teeth. Dental simulators have been created with different materials [12,13]. Three-dimensional-printed resin simulators were first developed for pedagogical purposes [14], and it was reported that their use had positive effects on students’ self-confidence [15]. Three-dimensional-printed simulators have not yet been used in comparison with human teeth during experimental mechanical tests.

This in vitro study was primarily designed to compare the fracture resistance of human mandibular molars, treated either with full pulpotomy or with root canal treatment and then prepared according to two designs of coronal preparation to be finally restored with an endocrown milled using either a composite or ceramic block. The second goal was to assess whether 3D-printed resin simulators could be a reliable alternative for human teeth during in vitro strength tests.

## 2. Materials and Methods

### 2.1. Samples and Study Design

**Figure 1:**
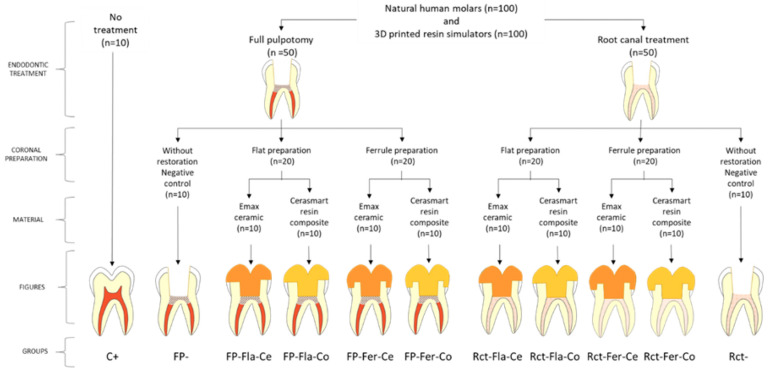
Study design. C+: sound teeth (positive control); FP−: teeth with FP and no restoration (negative control for FP-treated teeth); FP-Fla-Ce: FP-treated teeth restored with a flat margin ceramic endocrown; FP-Fla-Co: FP-treated teeth restored with a flat margin composite endocrown; FP-Fer-Ce: FP-treated teeth restored with a ceramic endocrown with 2 mm circumferential ferrule; FP-Fer-Co: FP-treated teeth restored with a resin composite endocrown with a 2 mm circumferential ferrule; Rct-Fla-Ce: root-canal-treated teeth restored with a ceramic endocrown with a flat margin; Rct-Fla-Co: root-canal-treated teeth restored with a resin composite endocrown with flat margin; Rct-Fer-Ce: root-canal-treated teeth restored with a ceramic endocrown with a 2 mm circumferential ferrule; Rct-Fer-Co: root-canal-treated teeth restored with a composite endocrown with a 2 mm circumferential ferrule; Rct−: teeth with RCT without restoration (negative control for root-canal-treated teeth). This study design was duplicated with 3D-printed resin simulators.

Human teeth (HT): Authorizations for using natural human teeth were obtained from patients treated in the dental department of the University Hospital of Clermont-Ferrand, France. One hundred and ten human mandibular molars, extracted for orthodontic or periodontal reasons, without caries, enamel cracks, or anatomical defects, were collected and kept in sodium chloride (NaCl 0.9%) solution. The teeth were randomly divided into eleven groups according to endodontic treatments, types of coronal preparation, and milling materials for endocrowns (Figure 1): 10 teeth were kept sound without any preparation; 50 teeth received root canal treatment; and 50 other teeth received full pulpotomy. In each subgroup of 50 teeth, 10 teeth were left without restoration after endodontic treatment (Rct or FP). The other 40 were divided into 4 groups of 10 according to the material used and the type of coronal preparation. 

The number of samples needed was estimated from a preliminary pilot study analyzing the fracture strength resistance between natural root-canal-treated teeth restored with flat margin endocrowns vs. those restored with 2 mm circumferential ferrule endocrowns. The mean strength value was higher for teeth restored with flat margin endocrowns (1630 ± 320.3 N versus 1076 ± 328.1 Newtons, respectively) (SD = 406.3). Calculations were based on this difference for a continuous criterion with independent values and indicated the need for at least 10 samples for each group (α = 5%; β = 15%; epiR package 0.9–30). 

The procedures for endodontic treatment and coronal preparation are detailed in Table 1. 

**Figure 2:**
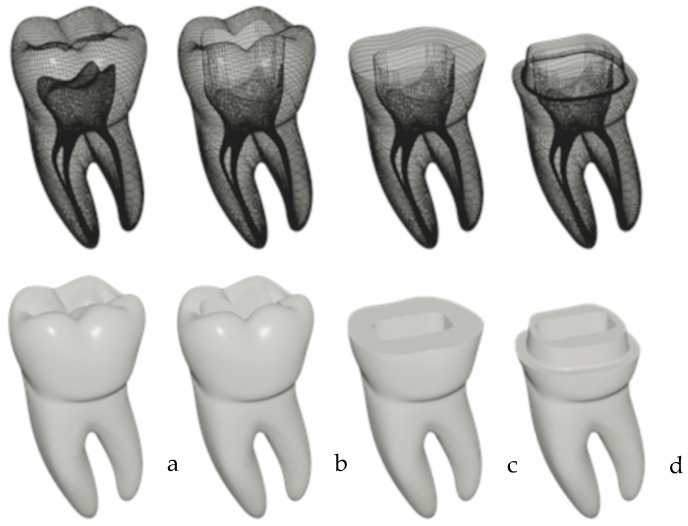
Three-dimensional representation of the different resin simulators. (**a**) resin simulator representing sound teeth; (**b**) resin simulator representing teeth with an access cavity used as negative control; (**c**) resin simulator representing teeth with a flat peripherical coronal preparation, and (**d**) resin simulator representing teeth with a 2 mm peripherical ferrule.

Resin simulators (RSs): One first lower left molar was created using 3D modeling using Blender software (v3.2.1, Blender Foundation) with one mesial root with two independent canals and one distal root with one central canal. Four versions of this model were then created, differing in the presence or absence of an endodontic access cavity and the type of crown preparation (Figure 2): 10 duplicates of the model without coronal preparation or an access cavity (Figure 2a), 20 duplicates with an access cavity (Figure 2b), 40 duplicates with flat peripherical coronal preparation (Figure 2c), and 40 duplicates with a 2 mm peripherical ferrule (Figure 2d). The simulators were manufactured using a STRATASYS™ Objet30 Prime 3D printer (Polyjet technology, Eden Pairie, MN, USA) with RGD525 resin, which provides the best mechanical characteristics. The simulators were built using multiple 28 µm photo-polymerized layers from root to crown. The surface finish was defined as glossy, which improves strength, and no post-curing protocol was used.

### 2.2. Study Criteria

The main study criterion was the fracture strength resistance given in Newtons. The secondary criterion was the type of fracture [16]: (i) a catastrophic fracture was below the cementoenamel junction and extended up to the root; (ii) a favorable fracture was one that was above the cementoenamel junction. The explicative factors were (i) the type of endodontic treatment, RCT or FP; (ii) the type of preparation for the endocrowns, with a 2 mm circumferential ferrule (Fer) or flat margin (Fla); and (iii) the material of the endocrowns, ceramic (IPS e.max CAD^®^, Ce, Ivoclar-Vivadent, Schaan, Liechenstein) or composite (Cerasmart^®^, Co., GC, Tokyo, Japan).

### 2.3. Loading Test 

All 110 human teeth and 110 3D-printed resin simulators were embedded individually in self-cured epoxy resin (U-Pol Plastikit, Wellingborough, UK). Each sample was placed in the fixture of a universal testing machine (Zwick Roell UTS 20K, Ars-Laquenexy, France). The compression tests were carried out with a 5 mm diameter stainless steel sphere placed on the middle of the occlusal face and according to a 45° angle to the long axis. The samples were loaded at a rate of 0.5 mm/min until fracture. A measurement was made automatically every 0.1 s. A 30% reduction in the load strength resulted in automatically stopping the recording. 

### 2.4. Statistical Analysis

The data were statistically analyzed with SPSS software (28.0.1.1, IBM Corp, USA). The distribution of each group was tested using a Shapiro–Wilk test. In the case where all groups had a normal distribution, the groups were compared using a one-way analysis of variance (ANOVA) with a Student–Newman–Keuls post hoc test. If one group showed an abnormal distribution, the samples were compared using a Kruskal–Wallis test. The distribution of the fracture type was compared with a chi-square test. The human teeth and resin simulator strength values were compared for each condition with a Mann–Whitney test. The equality of variance between the RS groups and HT groups was compared with Levene’s test.

## 3. Results

### 3.1. Strength Test Values 

**Figure 3:**
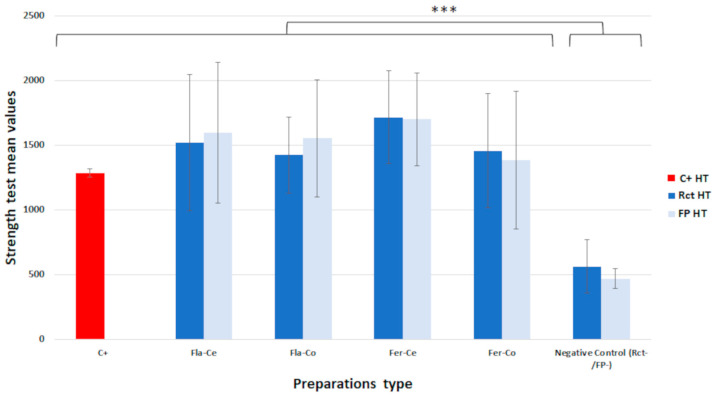
Comparison of strength test mean values assessed for human teeth according to the type of endodontic treatment, the type of coronal preparation, and the materials used for endocrown milling (Student–Newman–Keuls post-ANOVA; ***: *p* < 0.001).

**Figure 4:**
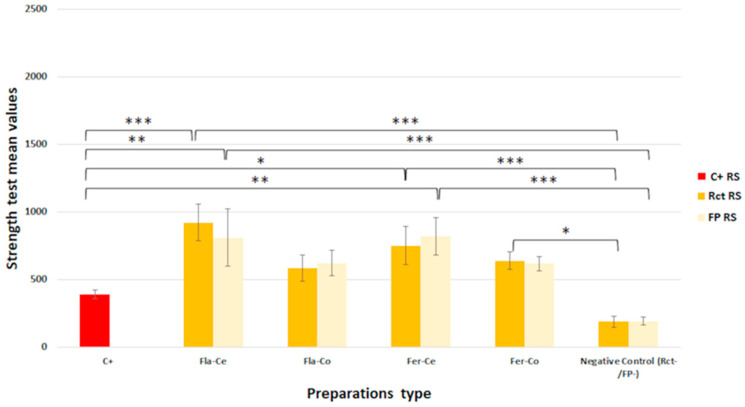
Comparison of strength test mean values assessed for resin simulators according to the type of endodontic treatment, the type of coronal preparation, and the materials used for endocrown milling (Kruskal–Wallis; *: *p* < 0.05, **: *p* < 0.01, and ***: *p* < 0.001).

The mean values of the maximum forces leading to the fracture of the sample groups are presented in Figure 3 and Figure 4.

The Shapiro–Wilk test validated a normal distribution for all the groups of human teeth but did not validate a normal distribution for the resin simulators. An analysis of variance (ANOVA) was performed for the human teeth groups and then a Kruskal–Wallis test was performed to compare the results for the groups of human teeth and resin simulators (Table 2). 

For the human teeth groups, the ANOVA test separated two homogeneous groups, the negative controls grouping the root-canal-treated teeth or full-pulpotomy-treated teeth without coronal restoration (Rct− and FP−) and all the other samples, i.e., the untreated sound teeth (positive control, C+) and the treated and restored teeth. The negative control groups showed significantly lower resistance than the untreated sound teeth or the teeth treated with coronal restoration.

The results of the Kruskal–Wallis test were different for the resin simulators in contrast with natural human teeth. The resin simulators with ceramic restorations and either endodontic treatment (Rct or FP) had greater resistance than the simulators without treatment and those without restoration. Untreated simulators (positive control, C+) had the same resistance as simulators with endodontic treatment without restoration (negative controls, Rct− and FP−). Simulators with composite restorations had the same fracture resistance as simulators without restoration except for those with composite restorations with circumferential ferrule preparation and in the case of Rct. 

### 3.2. Fracture Type 

Amongst the 110 human teeth, 36 had favorable fractures. The distribution of the type of fracture depending on the different groups is visible in Table 3. 

The type of fracture was always less favorable for treated teeth than for sound teeth. The type of fracture in all the groups of treated teeth was the same as that in the negative control groups except for that in the group of teeth treated with pulpotomy and flat margin ceramic endocrowns (FP-Fla-Ce), which was always catastrophic. 

Amongst the 110 resin simulators, 3 had favorable fractures. The distribution of the type of fracture depending on the different groups is visible in Table 4. 

The type of fracture was catastrophic for resin simulators regardless of the material, the preparation, or the endodontic treatment. 

## 4. Discussion

To our knowledge, this is the first work assessing the in vitro mechanical behavior of molars treated either with pulpotomy or root canal treatment and the first one that has demonstrated that the mechanical behavior of resin simulators does not reproduce that of human teeth. For human teeth, pulpotomy and root canal treatment had similar impacts on the strength resistance and the type of tooth fracture, regardless of the composition of the milling material and the type of peripheral limit. These results suggest that indications to use endocrown restoration should not be based on the mechanical resistance of endodontically treated teeth. Other parameters such as the pulpal diagnosis, the risk factors for occlusal and proximal wear, and the ferrule effect should be considered. Finally, the interest of the ferrule effect should be re-examined, at least with the objective of improving the mechanical resistance of endocrowns.

Pulpal diagnosis should be performed first to indicate either pulpotomy or root canal treatment. Full pulpotomy has been indicated for the treatment of reversible pulpitis associated with a carious lesion or a healthy pulp after traumatic pulp exposure in primary teeth and immature permanent teeth [17]. In mature permanent teeth, it was used as an emergency procedure before root canal treatment in teeth with irreversible pulpitis [18]. During the last decade, a better understanding of pulpal biology and the development of bioactive materials has led to reinvestigating pulpotomy as a definitive treatment for vital mature permanent teeth needing root canal treatment due to inflammatory conditions. Pulpotomy and root canal treatment demonstrate comparable and effective postoperative pain relief since the pulpotomy procedure provides a significant gain in time compared with root canal treatment [19]. Pulpal necrosis or the infection of one or more canals are contraindications for performing a pulpotomy. Many recent studies have shown that the quality of coronal restoration, its sealing, and the delay before its implementation are key factors for the success of a pulpotomy [20,21,22]. A better tooth survival rate is observed if the restoration is carried out within two days of pulpal exposure, thus emphasizing the need to limit the number of treatment sessions and the time between them [23]. Thus, when pulpotomy is biologically indicated and endocrown restoration can be performed within a short time, this treatment can be offered to patients without loss of chance from a mechanical point of view for the tooth compared with root canal treatment. Moreover, patients spend less time in the chair, and the iatrogenic risks inherent to root canal treatment (instrumental fracture, stripping, etc.) can be avoided. This is particularly true in molars where iatrogenic perforations are more frequent, especially if they have significant curvatures [24]. Today, the use of posts is limited because it does not increase tooth resistance and is indicated only in case of high loss of dental tissue to increase the retention of restorations [25]. Furthermore, the use of posts in the posterior sector is considerably reduced, and an endocrown [26] or overlay with a composite build-up [27] is proposed for a severely damaged tooth. In this respect, the indication for pulpotomy treatment is no longer limited.

The use of the WaveOne Primary file for the endodontic treatments performed in this study is justified by its ease of use (single instrument) and the lower risk of instrumental fracture and root microcracks via its reciprocal movement. Studies have shown that reciprocating motion induces lower tensile and compressive stress in the flexed region of the instrument, thus providing greater fatigue resistance compared with continuous rotative movement [28,29]. High-taper instruments, such as the WaveOne Primary (25/0.08), can contribute to deeper infected-tissue removal, achieving the appropriate irrigant penetration level [30], and a recent systematic review concluded that it is not clear whether a difference in taper angle can determine differences in root fracture resistance; therefore, it is not possible to provide clinical recommendations regarding the use of endodontic instruments with a high (≥6%) or a low (<6%) taper [31].

This study showed that the resistance strength of the restored teeth was similar when comparing composite and ceramic endocrowns. This point has been discussed in previous studies and remains a subject of interest. Finite element studies have shown less stress generated for ceramic [32,33,34,35] and composite [36,37,38] endocrowns. In contrast, in vitro studies have reported better resistance with less favorable fractures for ceramic endocrowns [16,39] than for composite endocrowns [16,40,41,42]. Moreover, indications for the use of ceramic rather than composite endocrowns should be considered with regard to the high rate of composite wear [43,44]. Clinicians are faced with a conflictual choice between ceramic and composite endocrowns. On the one hand, a composite material leads to fractures of the endocrown, allowing the tooth to be re-restored. On the other hand, ceramic material offers a sustainable solution to restore proximal and occlusal contacts. An original two-piece combination of one build-up made of a resin composite in the pulp chamber and a ceramic overlay was proposed. It was demonstrated that the mechanical resistance of the two-piece restorations was similar to that of ceramic endocrowns [45]. For teeth treated with pulpotomy, a layer of material used for pulp capping is always present in the chamber volume and more or less fills all the pulp volume, as performed in a build-up. In such situations, the question is whether the pulp-capping material could have sufficient biomechanical qualities to be considered as a core build-up. Several studies have reported the use of mineral trioxide aggregate (MTA) material, calcium-enriched mixture (CEM), and Biodentine^®^ for pulp capping after full pulpotomy. It was demonstrated that Biodentine^®^ has a higher resistance value to compressive strength than CEM or MTA, which reaches the resistance of dentine [46] but remains lower than that of a composite [47]. In order to replace a composite core with a Biodentine^®^ core, it is necessary to verify whether it is possible to bond a ceramic endocrown on Biodentine^®^. It was shown that regarding resistance to shear, the bond strength is lower for the Biodentine^®^/ceramic interface than for the composite/ceramic interface [48]. To improve the resistance of the Biodentine^®^/ceramic interface, some authors suggest applying a composite layer between the Emax overlay and the Biodentine^®^ core. The bonding system with 10-MDP seems to give a better interface quality than other bonding systems [49]. Further studies are necessary to investigate the quality of the interface between both materials used for endocrowns and a core build-up and, particularly, to test whether the quality of the interface also depends on the type of ceramic used for endocrowns. 

The present study showed that there is no ferrule effect in the case of preparations for endocrowns. However, when the teeth were prepared for full crowns, the ferrule effect was considered an important criterion for survival prognosis. This effect was recently discussed in a systematic review [2]. In vitro studies showed a protective effect of the ferrule in relation to the fracture [50,51,52], and in vivo studies showed a very high survival rate in which failures were due to the debonding of the endocrown and not to the fracture [7,8]. The advantages of preparations with a ferrule were related to the height of the ferrule and the bevel of the margin. A recent finite element study proposed to perform a 20° bevel, which would produce the same effect as a conventional ferrule [37]. In the present study, a 2 mm ferrule was chosen because it was reported in the literature that this value was necessary to protect teeth with full crowns from catastrophic failure. Although one study showed that a height of 1 mm was a better value for an endocrown restoration [51], another study showed a contradictory result, with better protection with a 2 mm ferrule than with a 1 mm ferrule [52]. In the present study, the presence of a ferrule had no effect on the fracture resistance, although the absolute resistance value was recorded for the group with a ceramic endocrown with a ferrule preparation (group Rct-Fer-Ce). Including a smaller number of groups with a focus on teeth treated with pulpotomy might increase the possibility of making these differences significant. 

This study demonstrated that 3D-printed resin simulators could not be used in place of human teeth for in vitro resistance studies. However, the variance of the mean strength value was lower for 3D-printed simulators than for human teeth. This suggests that the modeling of resin simulators allows controlling the variability of natural human teeth size and shape. Thus, the modeling of simulators could be interesting in experimental studies in which new materials with mechanical behaviors close to those of enamel and dentin are used in printers.

One limitation of this study is the absence of artificial aging. Given the large number of conditions tested in this study, the addition of thermocycling would have doubled the number of teeth needed. However, collecting teeth is a complex part of this type of study in a health care system that encourages the conservation of teeth and where, in the concept of minimally invasive surgery, the extraction of complete teeth is rare. Another limitation is that this study was conducted on mandibular molars and cannot be generalized for other posterior teeth, particularly the maxillary premolar, which is a tooth at high risk of fracture. For certain in vitro studies, for example, the use of a fiber post increased the fracture resistance of maxillary premolars [53], which is not the case for the other posterior teeth [25]. A finite element study suggested that endocrowns could be the best restoration for maxillary premolars [54]. Further studies could be designed to investigate the effect of the type of teeth on endocrown fracture resistance. Finally, one can question the relevance of comparing the in vitro fracture resistance of natural teeth treated either with root canal treatment or full pulpotomy. The starting hypothesis was the fact that, in vivo, dentin moisture decreases Young’s modulus of radicular dentine [55] and, in turn, acts as a protective factor against fracture. However, in in vitro studies, not all avulsed teeth contained pulp tissue, and one cannot expect any difference in fracture resistance between groups with root canal treatment or pulpotomy. In vivo long-term follow-up studies would therefore be necessary to estimate the impact of the presence of vital pulp tissue on tooth strength. In such studies, other parameters could act as confounding factors, as the extent of the tissue damage would be greater in teeth with root canal treatment than in teeth with pulpotomy.

## 5. Conclusions

Pulpotomy and root canal treatment had similar impacts on the strength resistance and the type of fracture of teeth restored with endocrowns, regardless of the composition of the milling material and the type of peripheral limit. Within the limits of this study, this work suggested that endocrowns can, therefore, be chosen as a restoration for teeth treated with pulpotomy. This study provides new perspectives for treating teeth with inflammatory pulpal disease in one single chairside session in combination with the use of CAD/CAM processing. Three-dimensional-printed resin simulators should not be used in place of human teeth for in vitro resistance studies because their behavior in fracture resistance tests (strength test values and type of fracture) is not comparable to that of human teeth.

## Figures and Tables

**Table 1:** Procedures for endodontic treatment and coronal preparation.

**Endodontic Treatments**	
Root canal treatment:	The pulp chamber was opened with a diamond bur (Komet, Gebrüder Brasseler, Lemgo, Allemagne). The access cavity was created with an Endo-Z bur (Dentsply Sirona, Bensheim, Germany). A no. 10 K file was used to determine the working length. The root canals were instrumented with a Niti file in reciprocating movement (Wave One Primary Dentsply Sirona, Bensheim, Germany). Irrigation was ensured with a 2.5% sodium hypochlorite solution. Obturation was performed with a zinc oxide eugenol sealer (Sealite, Pierre Roland, Merignac, France) and an appropriate Gutta Percha point for the Niti file (Wave One Primary Dentsply Sirona, Bensheim, Germany). Root canals were obturated via thermomechanical compaction. For the 3D-printed simulators, the treatment procedure was the same, except for the chamber opening as the simulators were printed with an access cavity.
Full pulpotomy:	After opening and creating the access cavity, a third coronary preparation was made with a Gates Glidden Drill no. 2 (Dentsply Sirona, Bensheim, Germany). Irrigation was carried out with a 2.5% sodium hypochlorite solution. Drying was performed with a cotton pellet. The bioactive material (Biodentine^®^, Septodont, Saint Maur des Fossés, France) was positioned in the pulp chamber and compacted with an amalgam plugger. An endo-stop was positioned on it to measure a 3 mm thickness of the obturation material.
**Coronal Preparations**	
Flat margin coronal preparation:	The depth of occlusal reduction was marked on the teeth with a specialized bur (Deep Marker, Komet, Gebrüder Brasseler, Lemgo, Germany). The margin was regularized with a diamond wheel bur (Komet, Gebrüder Brasseler, Lemgo, Germany).
Peripherical ferrule preparation:	After the flat margin preparation, the deep marker was reused to mark a 2 mm height on each face, and a 2 mm guided bur was used to perform the ferrule preparation.
**Endocrown Materials and Protocols**	
Restoration design and milling:	Teeth were mounted in a master model and scanned with Cerec Primescan (Dentsply Sirona Dental System, Benscheim, Germany). The restorations were designed with the biocopy function from a reference tooth from a Frasaco model (Frasaco Gmbh, Tettnang, Germany). All endocrowns were milled with a Cerec InLab MCXL milling unit (Dentsply Sirona Dental System, Benscheim, Germany). The ceramic restorations were crystallized using a Programat CS furnace (Ivoclar Vivadent, Schaan, Lichtenstein). For the 3D simulators, the biocopy modelling function of the Cerec Primescan system was applied using the simulator of untreated teeth as the model.
Emax adhesive protocol:	Endocrown intrados were etched for 20 s using 9% hydrofluoric acid (Porcelain Etch, Ultradent, South Jordan, USA). After rinsing (30 s) and drying, a silane coupling agent was applied for 1 min (Monobond Plus, Ivoclar Vivadent, Schaan, Lichtenstein). Teeth or simulators were etched with 35% orthophosphoric acid (CyberEtch, Henry Schein, Joué-les-Tours, France), and ExciTE (Ivoclar Vivadent Schaan, Lichtenstein) bonding was applied with a microbrush. Cementation was performed with a Variolink Esthetic DC (Ivoclar Vivadent Schaan, Lichtenstein) under digital pressure. A light cure was performed 2 times for 20 s.
Cerasmart (GC Tokyo, Japan) adhesive protocol:	Endocrown intrados were etched for 20 s using 9% hydrofluoric acid (Porcelain Etch, Ultradent, South Jordan, USA). After rinsing (30 s) and drying, a primer agent was applied (G Multi Primer, GC, Tokyo, Japan). Teeth or simulators were etched with 35% orthophosphoric acid (CyberEtch, Henry Schein, Joué-les-Tours, France), and G-Premio BOND bonding (G Multi Primer, GC, Tokyo, Japan) was applied with a microbrush, and followed by waiting for 10 s, drying for 5 s, and light curing for 10 s. Cementation was performed with a G-CEM LinkForce (GC Tokyo, Japan) under digital pressure. A light cure was performed twice for 20 s.

**Table 2:** Comparison of strength test values according to the type of endodontic treatment, the type of preparation, and the materials used for endocrowns for the human teeth and the resin simulators (Kruskal–Wallis test, NS: Not Significant).

		Human Teeth (*p*)										
		C+	FP-Fla-Ce	FP-Fla-Co	FP-Fer-Ce	FP-Fer-Co	Rct-Fla-Ce	Rct-Fla-Co	Rct-Fer-Ce	Rct-Fer-Co	FP−	Rct−
Resin simulators (*p*)	C+	-	NS	NS	NS	NS	NS	NS	NS	NS	0.003	0.009
	FP-Fla-Ce	0.008	-	NS	NS	NS	NS	NS	NS	NS	<0.001	<0.001
	FP-Fla-Co	NS	NS	-	NS	NS	NS	NS	NS	NS	<0.001	<0.001
	FP-Fer-Ce	0.001	NS	NS	-	NS	NS	NS	NS	NS	<0.001	<0.001
	FP-Fer-Co	NS	NS	NS	NS	-	NS	NS	NS	NS	0.001	0.003
	Rct-Fla-Ce	<0.001	NS	NS	NS	NS	-	NS	NS	NS	<0.001	<0.001
	Rct-Fla-Ce	NS	NS	NS	NS	NS	0.05	-	NS	NS	<0.001	0.002
	Rct-Fer-Ce	0.014	NS	NS	NS	NS	NS	NS	-	NS	<0.001	<0.001
	Rct-Fer-Co	NS	NS	NS	NS	NS	NS	NS	NS	-	<0.001	<0.001
	FP−	NS	<0.001	NS	<0.001	NS	<0.001	NS	<0.001	0.024	-	NS
	Rct−	NS	<0.001	NS	<0.001	NS	<0.001	NS	<0.001	0.02	NS	-

**Table 3:** Comparison of distribution of the type of fracture in each group for the human teeth (chi-square test, NS: Not Significant).

Group	Favorable Fracture (n)	Catastrophic Fracture (n)	Significance Compared with the Positive Control (*p*)	Significance Compared with the Negative Control (FP− or Rct−) (*p*)
C+	10 (100%)	0		
FP−	5 (50%)	5 (50%)	<0.01	
FP-Fla-Ce	0	10 (100%)	<0.001	<0.01
FP-Fla-Co	3 (30%)	7 (70%)	<0.01	NS
FP-Fer-Ce	1 (10%)	9 (90%)	<0.001	NS
FP-Fer-Co	3 (30%)	7 (70%)	<0.01	NS
Rct−	4 (40%)	6 (60%)	<0.01	
Rct-Fla-Ce	2 (20%)	8 (80%)	<0.001	NS
Rct-Fla-Ce	3 (30%)	7 (70%)	<0.01	NS
Rct-Fer-Ce	2 (20%)	8 (80%)	<0.001	NS
Rct-Fer-Co	3 (30%)	7 (70%)	<0.01	NS
Total	36 (32.7%)	74 (67.3%)		

**Table 4:** Comparison of distribution of the type of fracture in each group for the resin simulators (chi-square test, NS: Not Significant).

Group	Favorable Fracture (n)	Catastrophic Fracture (n)	Significance Compared with the Positive Control (*p*)	Significance Compared with the Negative Control (FP− or Rct−) (*p*)
C+	0	10 (100%)		
FP−	0	10 (100%)	NS	
FP-Fla-Ce	0	10 (100%)	NS	NS
FP-Fla-Co	1 (10%)	9 (90%)	NS	NS
FP-Fer-Ce	0	10 (100%)	NS	NS
FP-Fer-Co	1 (10%)	9 (90%)	NS	NS
Rct−	0	10 (100%)	NS	
Rct-Fla-Ce	0	10 (100%)	NS	NS
Rct-Fla-Ce	1 (10%)	9 (90%)	NS	NS
Rct-Fer-Ce	0	10 (100%)	NS	NS
Rct-Fer-Co	0	10 (100%)	NS	NS
Total	3 (2.7%)	107 (97.3%)		

## Data Availability

Data are available upon request.
